# Impact of Education on Trauma Patients’ Handover Quality; a Before-After Trial

**Published:** 2019-01-27

**Authors:** Ali Shahrami, Masoomeh Nazemi-Rafi, Hamidreza Hatamabadi, Afshin Amini, Mahammad Haji Aghajani

**Affiliations:** 1 Department of Emergency Medicine, Imam Hossein Hospital, School of Medicine, Shahid Beheshti University of Medical Sciences, Tehran, Iran.; 2 Emergency Department, Shahid Bahonar Hospital, School of Medicine, Kerman University of Medical sciences, Kerman, Iran.; 3 Cardiology Department, Imam Hossein Hospital, School of Medicine, Shahid Beheshti University of Medical Sciences, Tehran, Iran.

**Keywords:** Clinical audit, emergency service, hospital, patient handoff, checklist, patient safety

## Abstract

**Introduction:**

Poor handover and inadequate transmission of clinical information between shifts cause a lot of problems in patient care and result in significant risks for physicians and patients. This study was designed to evaluate the impact of education and application of handover checklist on trauma patients’ handover quality.

**Methods:**

In this before-after trial, handover process of trauma patients in an educational hospital was evaluated before and after education and application of a handover checklist, abbreviated as “WHO MISSED IP?”, using a questionnaire that consisted of 10 necessary items, which should be delivered during handover of trauma patients. A total score of 10 was considered for each patient handover, the score 10 out of 10 indicating that all 10 important pieces of patient information were correctly delivered.

**Results:**

52 pre and post-intervention handover sessions were evaluated (438 patients). Prior to intervention, 18% of patients were not delivered to the next shift, most of which were in the night shift handover (p < 0.001). From the pre-intervention to the post-intervention period, significant improvements were detected in all items except for diagnosis and consulting items. The mean duration of handover changed from 1.22 ± 0.24 minutes to 1.58 ± 0.23 minutes after intervention (p < 0.01). In the pre-intervention period, the score equal or greater than 9 was observed in 7.5% of patients, while after intervention, 63.6% of patients had score ≥ 9 regarding complete handover (p < 0.01).

**Conclusion:**

Based on the findings of the present study, teaching handover standards and application of handover checklist could be helpful in improving the quality of information delivery between emergency medicine residents and improve trauma patients’ handover indices.

## 1. Introduction

Traumatic injuries are an international issue that affects all aspects of life (1). Unfortunately, in developing countries, little attention has been paid to this problem and trauma is the first cause of death and a main cause of disability of active population.

Emergency department (ED) is a critical area in the healthcare field. In this territory, most of trauma patients are in danger, not only because of the injury, but due to the communication errors between hospital staff (2, 3). Weak communication is known as a major factor responsible for 24% of missed diagnoses in ED (2, 4). In trauma patients, more than 10 percent of deaths could be prevented by eliminating theses errors (5). 

In order to reduce the errors, the Joint Commission has implemented a standardized method for patients’ handover. However, the complexity and diversity of healthcare makes it impossible to apply a single standard protocol for all hospital sectors. ED has the highest number of patient deliveries; however, in this department, there is still a lack of standard protocols for patients handover (6, 7), especially for trauma patients (3). In a study on trauma patient handover from ED to intensive care unit, it has been demonstrated that a checklist alone was insufficient. They emphasized the importance of training and standardizing the handover process (3).

Based on the above-mentioned points, this study was designed to evaluate the impact of education and application of handover checklist on trauma patients’ handover quality.

## 2. Methods:

***2.1. Study Design and setting***

This before-after trial was conducted on the handover process of trauma patients in the emergency department of Imam Hossein Hospital, an educational hospital affiliated to Shahid Beheshti University of Medical Sciences, Tehran, Iran. The pre-intervention survey took place between 31 March and 9 May 2016, while the post-intervention period was performed between 16 May and 24 June. The protocol of study was approved by Ethics Committee of Shahid Beheshti University of Medical Sciences.

***2.2. Participants***

All emergency medicine residents present during the handover period of trauma patients at the morning and night shifts participated. There was not any limitation regarding the residents’ age and sex and also trauma severity of patients. 

***2.3. Data gathering***

A questionnaire that consisted of 10 necessary items, which should be delivered during handover of a trauma patient, including patient’s identification, mechanism of trauma, injury/s, vital signs, findings of history taking and clinical examination, para-clinical findings (imaging and laboratory findings), therapeutic measures, results of consultation with other disciplines, diagnosis, and plan were filled out for handover of all patients before and after education and application of a designed checklist for trauma patients’ handover. A total score of 10 was considered for each patient handover, the score 10 out of 10 indicating that all 10 important pieces of patient information were correctly delivered.

A senior emergency medicine resident imperceptibly observed and evaluated the handover process of patients. She was also responsible for data gathering.

At the mentioned trauma unit, handovers were performed verbally along with taking notes, among second-year residents. The morning and night handover sessions had little shift overlap, and were from 8-8:30am and 8-8:30pm. 

***2.4. Developing handover checklist***

Reviewing the related medical literature, observing strong and weak points of real time handover of trauma patients, and with the guidance of experts in the field, the most important and necessary items in the handover process were extracted and used for developing a specific checklist. This mnemonic checklist was introduced on the basis of “WHO MISSED IP?” phrase with “Who” (patient ID as patient's name, sex, age and pre-injury health status), “M” Mechanism of trauma, “I” Injury (suspected or sustained), “SS” Sign & Symptom (containing observations and monitoring), “E” Evaluation (Imaging, laboratory, etc.), “D” Diagnosis, “I” Intervention (therapy and consulting), “P” Plan for patient management, and "?" giving an opportunity to question in the case of any ambiguity. 

***2.5. Intervention***

After the first phase (evaluation of patient handover before education and checklist application) and during the weekly conference, the correct ways of handover process were taught to the same residents. After familiarizing these residents with the standards of patient handover and introducing the developed checklist, handover audits were carried out again with the same residents and under the same conditions using the same questionnaire. 

***2.6. Outcome Measures***

The main outcome of this study was evaluating the changes of handover process of trauma patients before and after education and application of checklist. 

***2.7. Statistical analysis***

Data of pre and post-intervention periods were analyzed and compared via SPSS 21 software. The data were analyzed using t test for quantitative variables and chi-square test or Fisher’s exact test for categorical variables. Findings were presented as mean ± standard deviation or frequency (%). P < 0.05 was considered as statistically significant.

## 3. Results:

26 handover sessions (13 day and 13 night shifts) during each of the pre-intervention and post-intervention phases (52 handover sessions in total) were evaluated (438 patients). Prior to intervention, 18% of patients were not handed over, most of which were in the night shifts. This measure was reduced to 6% after intervention (p < 0.001). Table 1 shows the situation of information delivery during handover process regarding the 10 studied items. From the pre-intervention to the post-intervention period, significant improvements were detected in all items of the questionnaire except for diagnosis and consulting items. The mean duration of handover changed from 1.22 ± 0.24 minutes to 1.58 ± 0.23 minutes after intervention (p < 0.01). In the pre-intervention period, the score equal or greater than 9 was observed in 7.5% of patients, while after intervention, 63.6% of patients had score ≥ 9 regarding complete handover (p < 0.01). Table 2 shows the range of scores obtained regarding complete handover before and after education and application of handover checklist.

## 4. Discussion:

Based on the findings of the present study, teaching handover standards and application of handover checklist could be helpful in improving the quality of information delivery between emergency medicine residents and improve trauma patients’ handover indices.

The main aim of each handover is flawless transmission of information (6). To achieve this goal, each hospital department must have its own handover policy. This policy should be chosen by identifying existing problems such as absence of key people, defects in the structure and time allocated to the transmission of information, and responsibility (7). 

In this study, we established a standardized method for transmitting data by examining the previous status and recognizing its problems and weaknesses. Then, after teaching this standardized method to the residents, we evaluated its efficiency in improving the handover quality. We used two methods, at the same time, to improve the handovers: 1- Designing a pattern for information transmission in the form of acronyms “WHO MISSED IP?” to recall items. 2- Transformation of verbal handover into a written one using the handover checklist. The results of this study showed that a standardized model for handover of trauma patients among emergency residents could lead to an increase in patients' safety by transmitting true and complete information.

Prior to intervention, a significant number of patients (18%) were not delivered to the next shift, most of which were in the night shift. The reason for missing those patients was that they were not present during the handover period because they had gone to the imaging or outpatient surgery units. After the educational program, with emphasis on the fact that all patients should be delivered even if they are temporarily not in the department (2), this rate reduced significantly and reached 6%.

**Table 1:** Prevalence of handover shortcomings before and after education and application of handover checklist

**Information**	**Before (n=199)**	**After (n=239)**	**P Value**
**Patient identification**	116 (58.3)	56 (23.4)	< 0.001
**Trauma mechanism**	45 (22.6)	18 (7.5)	< 0.001
**Injury/s**	136 (68.3)	89 (37.2)	< 0.001
**History and examination**	115 (57.8)	47 (19.7)	< 0.001
**Vital signs**	136 (60.4)	89 (39.6)	< 0.001
**Para-clinic findings**	68 (34.2)	43 (18.0)	< 0.001
**Diagnosis**	15 (7.5)	29 (12.1)	0.06
**Therapeutic measures**	75 (37.7)	24 (10.0)	< 0.001
**Consult with other disciplines **	6 (3.0)	13 (5.4)	0.78
**Plan **	47 (23.6)	34 (14.2)	0.01

Data are presented as number (%).

**Table 2:** Rage of score gained regarding complete handover before and after education and application of handover checklist

**Score range**	**Before (n=199)**	**After (n=239)**	**P valve**
**Very poor (1-2)**	3 (1.5)	0 (0.0)	< 0.001
**Poor (3-4)**	39 (19.6)	7 (2.9)	
**Fair (5-6)**	85 (42.7)	27 (11.3)	
**Good (7-8)**	57 (28.6)	53 (22.2)	
**Very good (9-10)**	15 (7.5)	152 (63.6)	

Data are presented as number (%).

After the training, despite the implementation of checklist application, and contrary to our expectations, in the handover of 45% of patients, the checklist was not used, most of which were at night (only 34% of patients in night and 94% in day were handed over with checklist). Residents declared that they did not use checklist due to the ED being crowded, especially at night. The increase in patient reception at night could be a challenge to providing care and continuity of patient information. Moreover, when there is a heavy workload, abbreviations of verbal handover would result in inadequate communication and information oblivion (8).

This study clearly demonstrated that the “WHO MISSED IP?” educational program and checklist improved not only the number of patients delivered, but also the quality of the transmission of information. Our results are in agreement with reports that emphasized the positive effects of education (9) and the mnemonic checklist (8, 10, 11) on improving the quality of the handovers. In this study, education and checklists were associated with proper information transfer (score higher than 9) rate improving from 25% to 94%, which indicates the impressive impact of the checklist on the quality of the handover.

The handover duration is one the parameters that scholars are interested in shortening its value, along with increasing the quality of handover. A study that was conducted on non-surgical patients in a specialist hospital showed that in the presence of a designed checklist, along with the increase in the quality, the duration of the handover was significantly reduced (a reduction of 22% from 99 ± 3.3 seconds to 77 ± 2.8 seconds per patient) (10). However, in our study, which was conducted on trauma patients, handover duration had increased significantly after education (from 73 ± 2.4 s to 96± 1.8 s per patient). This parameter was higher than the 73 to 92 seconds duration reported in ED (12), which could be due to the high information deficit before the intervention. On the other hand, the handover duration can also directly affect the content of the handover (13). Therefore, the education and standard model increased the transmission of information and, as a result, increased the handover duration.

In this study, to improve the efficiency of the handovers, the item “?” was designed to provide the opportunity of clarifying the ambiguous points by encouraging the receiving emergency residents to ask questions about handover (2).

Due to the timing of the night handover being at the peak of the trauma emergency, it is suggested to consider a scheduled shift overlap, so that the physicians handing over the patients have enough time to complete transfer of patient information (2). In addition, in order to improve the quality of the handover, it is recommended to train residents (for example, annually) about handover standards. Also, the use of other handover methods, such as the electronic method, should be considered in order to maintain patient safety during this repeated and potentially risky process.

Suitable handover is not achieved by chance. It needs dedication of time, education, cooperation and alertness of all team members. We demonstrated that use of a mnemonic checklist and involvement of junior residents correlated with significant reduction in handover shortcomings. Thus, the handover style should be specifically designed based on the requirements of specific units. 

***Limitations***

This study was subject to limitations. The Hawthorne effect could affect the post-intervention phase. 

## 4. Conclusion:

Based on the findings of the present study, teaching handover standards and application of a handover checklist could be helpful in improving the quality of information delivery between emergency medicine residents and improve trauma patients’ handover indices.

## Author Contribution:

• Study conception and design: Shahrami, Hatamabadi, Amini, Nazemi-Rafi

• Acquisition of data: Nazemi-Rafi

• Analysis and interpretation of data: Nazemi-Rafi

• Drafting of manuscript: Nazemi-Rafi, Shahrami

• Critical revision: Hatamabadi, Amini

## Authors' ORCIDs

Ali Shahrami: 0000-0002-3001-6762

Masoomeh Nazemi-Rafi: 0000-0003-4782-2901

Hamidreza Hatamabadi: 0000-0002-9085-8806

Afshin Amini: 0000-0001-8928-8212
